# Genome-wide variation in the Angolan Namib Desert reveals unique pre-Bantu ancestry

**DOI:** 10.1126/sciadv.adh3822

**Published:** 2023-09-22

**Authors:** Sandra Oliveira, Anne-Maria Fehn, Beatriz Amorim, Mark Stoneking, Jorge Rocha

**Affiliations:** ^1^ CIBIO, Centro de Investigação em Biodiversidade e Recursos Genéticos, InBIO Laboratório Associado, Campus de Vairão, Universidade do Porto, 4485-661 Vairão, Portugal.; ^2^ Department of Evolutionary Genetics, Max Planck Institute for Evolutionary Anthropology, Leipzig 04103, Germany.; ^3^ Computational and Molecular Population Genetics, Institute of Ecology and Evolution, University of Bern, 3012 Bern, Switzerland.; ^4^ Biopolis Program in Genomics, Biodiversity and Land Planning, CIBIO, Campus de Vairão, 4485-661 Vairão, Portugal.; ^5^ Departamento de Biologia, Faculdade de Ciências, Universidade do Porto, 4099-002 Porto, Portugal.; ^6^ Université Lyon 1, CNRS, Laboratoire de Biométrie et Biologie Evolutive, UMR 5558 Villeurbanne, France.

## Abstract

Ancient DNA studies reveal the genetic structure of Africa before the expansion of Bantu-speaking agriculturalists; however, the impact of now extinct hunter-gatherer and herder societies on the genetic makeup of present-day African groups remains elusive. Here, we uncover the genetic legacy of pre-Bantu populations from the Angolan Namib Desert, where we located small-scale groups associated with enigmatic forager traditions, as well as the last speakers of the Khoe-Kwadi family’s Kwadi branch. By applying an ancestry decomposition approach to genome-wide data from these and other African populations, we reconstructed the fine-scale histories of contact emerging from the migration of Khoe-Kwadi–speaking pastoralists and identified a deeply divergent ancestry, which is exclusively shared between groups from the Angolan Namib and adjacent areas of Namibia. The unique genetic heritage of the Namib peoples shows how modern DNA research targeting understudied regions of high ethnolinguistic diversity can complement ancient DNA studies in probing the deep genetic structure of the African continent.

## Teaser

DNA research from present-day populations deemed uncontactable or extinct helps probe the deep genetic structure of Africa.

## INTRODUCTION

It is generally accepted that the precolonial genetic diversity of southern Africa resulted from the sequential layering of at least three different sets of peoples with contrasting genetic, linguistic, and livelihood profiles: (i) an early occupation by the ancestors of foragers now speaking languages of the Kx’a and Tuu families (*1*–*3*); (ii) a more recent dispersal [~2 ka (thousand years) ago] of Late Stone Age pastoralists from East Africa, speaking languages of the Khoe-Kwadi family (*4*, *5*); and (iii) a subsequent arrival (~1.5 to 1.8 ka ago) of early farmer groups tracing their origins to West-Central Africa, who speak Bantu languages and rely to various degrees on agricultural and pastoral lifeways (*6*–*9*).

Previous studies have shown that autochthonous southern African foragers are among the most deeply divergent human populations and can be broadly subdivided into three major genetic subgroups associated with the northern, central, and southern Kalahari (*2*, *10*–*13*). Linguistically, the northern Kalahari is home to speakers of the Ju subgroup of Kx’a, while the central and southern Kalahari are mainly linked to the Taa and !Ui branches of Tuu, respectively (fig. S1, A and B) (*14*). Beyond the Kalahari Basin, ancestry related to southern African foragers has also been detected in contemporary populations from Zambia and eastern Africa (*2*, *15*), as well as in ancient DNA recovered from individuals who lived in Malawi between 8.1 and 2.5 ka ago (*16*, *17*). These findings indicate that foraging populations carrying a genetic ancestry related to modern Kx’a and Tuu speakers were more widespread in the past, before being absorbed or extinguished by incoming food producers.

The expansion of Bantu-speaking peoples, in particular, is known to have affected areas showing traces of previous forager occupation and might have played a major role in narrowing the distribution of foraging populations (fig. S1D) (*1*, *6*, *8*). Nevertheless, despite well-known cases of Bantu-forager admixture, most Bantu groups retain their cultural identity and are genetically closer to other Bantu speakers than they are to neighboring non-Bantu populations (*8*, *18*).

In contrast with the Bantu expansion, available data from groups speaking languages from the Khoe branch of Khoe-Kwadi suggest that the pastoral dispersal from East Africa associated with this language family was strongly shaped by complications arising from areal contact, admixture, and diffusion (*2*, *3*, *11*, *12*, *19*). Despite sharing various amounts of a genetic ancestry related to eastern Africa (*3*, *16*, *20*), Khoe groups include both pastoralist and foraging communities who have substantial genetic contributions from autochthonous southern African and Bantu-speaking populations (*2*, *12*). Because of this fragmented distribution of genetic and cultural makeups, the population landscape of southern Africa can only be fully understood by adopting a bottom-up approach that takes into account the complexity of the multiple contact scenarios involving Khoe-Kwadi speakers in different regions. Until now, the best studied areas are the Central Kalahari—home of Kalahari Khoe speakers—and, albeit to a lesser extent, the southern and western Kalahari Basin fringe historically occupied by Khoekhoe speakers (fig. S1C). In contrast, little is known about southwestern Angola. Here, the isolated Kwadi branch was spoken until the mid–20th century by a group known as the Kwepe who dwelt in the Angolan Namib Desert, close to the Kuroka River mouth (*21*).

Although both the Kwadi language and speech community were thought to be extinct, we were able to locate a group self-identifying as Kwepe who still live along the Kuroka River, in areas close to their originally reported location (fig. S2) (*22*–*24*). Despite the Kwepe’s recent shift to the Bantu language Kuvale (*25*), we identified the descendants of the Kwadi speakers recorded in 1965 by the linguist Ernst Westphal (*26*) and found two women who still remembered a considerable amount of the now extinct language’s lexicon and grammar. The present-day Kwepe are small stock herders who are surrounded by an array of dispossessed groups also inhabiting the Kuroka River Basin, known as Kwisi, Twa, and Tjimba (fig. S2). These three communities have been considered to descend from a distinct layer of pre-Bantu foragers, who occupied southern Africa along with the ancestors of Kx’a and Tuu speakers, and whose original language and culture have been lost (*27*, *28*). Regardless of their origins, the Kwepe and the other peoples from the Kuroka River Basin form a cluster of marginalized populations, sharing the languages and cultural habits of the Bantu-speaking Kuvale and Himba, who constitute part of the Herero pastoral tradition of southwestern Africa and represent the socially dominant force in the Angolan Namib (*27*, *29*). As they presently display a strong cultural and socioeconomic dependence on their dominant pastoral neighbors, the Kwepe, Kwisi, Twa, and Tjimba are best described as peripatetic communities, i.e., small-scale, highly mobile groups of low social status, who provide goods and services to others (*30*).

**Fig. 1.**
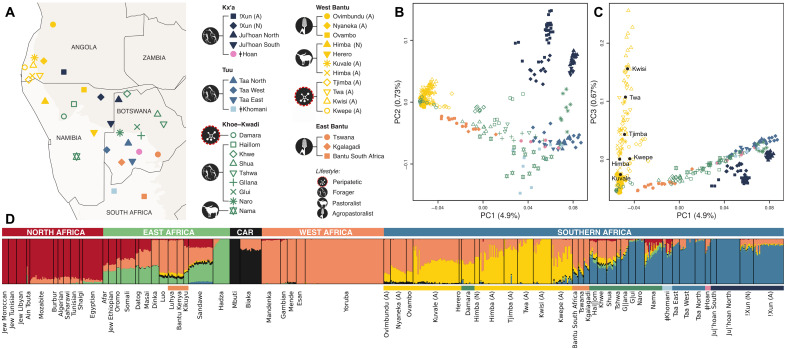
Population structure in southern Africa. (**A**) Map showing approximate sample locations. The lightest background color shows the desert and xeric shrublands biome. All samples from Angola are marked with “(A)” in the legend, while samples from the same ethnic group collected in Namibia are marked with “(N).” (**B** and **C**) PCA of the populations shown in (A). Plots of PC1 versus PC2 (B) and PC1 versus PC3 (C), with the percentage of variance explained by each component indicated in parentheses. The centroids for each group from the Angolan Namib are shown by black dots. (**D**) ADMIXTURE analysis for *k* = 6 (*k* with the lowest cross-validation error) of an extended dataset of 63 African populations (fig. S3 and tables S1 and S2). The horizontal color bar below the ADMIXTURE barplot indicates the language group. CAR, Central African Rainforest.

Motivated by the highly diverse population landscape of the Angolan Namib, we hypothesize that extant populations from this area may preserve part of the ancestry of the Kwadi, as well as genetic traces of vanished forager populations inhabiting southwestern Africa before the Bantu expansion. Here, we generated genome-wide data from 208 individuals belonging to nine ethnic groups from the Angolan Namib and surrounding areas, subsisting on foraging (!Xun), peripatetic (Kwepe, Kwisi, Twa, and Tjimba), pastoral (Himba and Kuvale), and agropastoral (Ovimbundu and Nyaneka) lifeways (text S1 and table S1). To contextualize our findings within the wider area of southern Africa and beyond, we further combined the data from Angola with other Africans previously genotyped on the same array (Fig. 1A, figs. S2 and S3, and table S2). Our results show that the descendants of the formerly Kwadi-speaking Kwepe and the other peripatetic groups from the Angolan Namib preserve a unique pre-Bantu genetic ancestry, highlighting the importance of southwestern Angola as a key area for understanding the peopling of southern Africa.

## RESULTS

In a principal components analysis (PCA) (*31*), the Angolan !Xun foragers are most similar to southern African groups speaking languages from the Ju branch of Kx’a, while all other sampled Angolan groups are closer to West Africans and to Bantu-speaking populations (Fig. 1B and fig. S3). Among those groups, the populations from the Angolan Namib display a notable substructure, forming a gradient of genetic differentiation best captured by principal component 3 (PC3), which stretches from the Kuvale and Himba cattle herders to the peripatetic Kwisi and Twa (Fig. 1C and fig. S4). In addition, the differentiation displayed in PC3 is correlated (*r* = 0.87; *P* < 0.001) with increasing amounts of an ancestry component revealed at *k* = 6 by the unsupervised population clustering approach implemented in ADMIXTURE (yellow in Fig. 1D and figs. S5 to S8) (*32*). This component overlaps with a previously identified “NW-Savannah” ancestry that was found to be especially common in northwestern Namibia among the Southwest Bantu–speaking Himba, Herero, and Ovambo (*11*, *12*). It is also predominant in the Khoekhoe-speaking Damara who are genetically very similar to the Herero and Himba (*2*, *24*) and probably adopted their language from Nama pastoralists with whom they had historically established a peripatetic-like association (text S1) (*30*). The varying proportions of this ancestry in southwestern Angola and northwestern Namibia seem to be broadly associated with subsistence strategy and socioeconomic status, being on average the lowest in the Nyaneka, Ovambo, and Ovimbundu agropastoralists (18 to 27%) and highest among the peripatetic Tjimba, Twa, and Kwisi (79 to 93%). An analysis of identity-by-descent (IBD) sharing (*33*) among these populations further indicates that between-group sharing of IBD segments is highest among the peripatetic communities (fig. S9).

As the southwestern Angolan pastoral scene is dominated by a highly hierarchized, caste-like matriclanic organization (text S1) (*24*), it is conceivable that the observed genetic structure was caused by drift and inbreeding associated with the marginalization of peripatetic communities. Alternatively, it is also possible that it reflects different levels of admixture with an unsampled or no longer existing population.

**Fig. 2.**
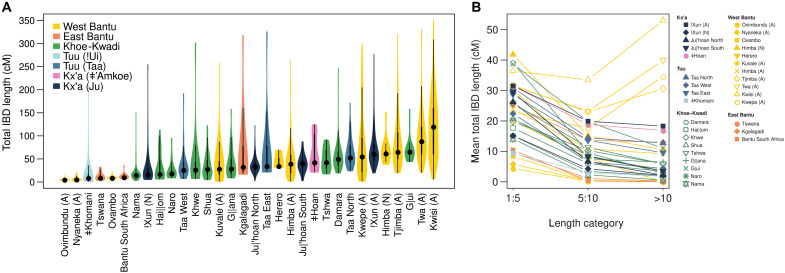
IBD sharing in southern Africa. (**A**) Violin plots showing the distribution of total IBD length (in centimorgans) shared within groups. The thick and thin black lines represent the interquartile range and the upper and lower adjacent values, respectively; the central dot shows the median. (**B**) Mean total IBD length for different length categories.

To investigate the role of genetic drift, we assessed the levels of genetic diversity in several populations from southwestern Angola and northwestern Namibia by computing for each group the total length of IBD (in centimorgans) segments shared between individuals (*33*) and the total length (in megabases) of runs of homozygosity (RoHs) per individual (*34*). We found that in southwestern Angola, the lowest and highest IBD and RoH lengths are found among agropastoralists and peripatetics, respectively (Fig. 2A and fig. S10A). Moreover, the peripatetic groups display significantly higher mean lengths of shared IBD segments above 10 cM than other southern African populations (Wilcoxon rank sum test, *W* = 0, *P* = 0.0016) (Fig. 2B). Estimates of effective population size (*Ne*) across time leveraging information from shared IBD segments (*35*) further reveal strong bottlenecks in peripatetic groups starting ~20 generations ago (fig. S11). Together, these results suggest that drift played a major role in the genetic differentiation of the Angolan communities with a lower socioeconomic status.

**Fig. 3.**
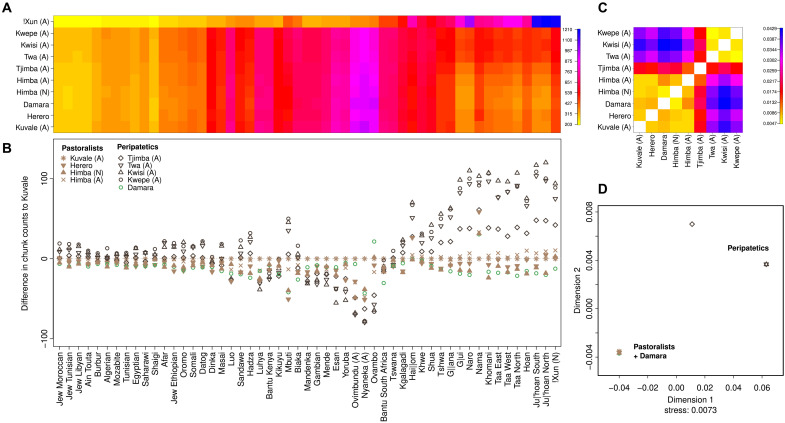
Chromopainter profiles for southwestern Angolan and northwestern Namibian groups. Bantu-speaking agropastoralists (Ovimbundu, Nyaneka, and Ovambo) are included as donors, together with more distantly related African groups. (**A**) Chromopainter coancestry matrix. The color gradient indicates the average number of DNA chunks a recipient group (*y* axis) copies from the donor populations [*x* axis in (B)]. (**B**) Differences between the number of DNA chunks copied by each recipient group from the donor populations in the *x* axis, and the number of DNA chunks copied by the Kuvale—used here as a baseline. (**C**) Average distance (TVDxy) between copying profiles. (**D**) Multidimensional scaling calculated on the TVDxy distances. The stress value (0.0073) indicates low distortion of population relationships by the two-dimensional plot (*75*).

We next used CHROMOPAINTER to investigate the genetic differentiation of the peripatetic groups while accounting for the effects of genetic drift (Fig. 3 and fig. S12). In CHROMOPAINTER, haploid genomes from recipient individuals are decomposed into chunks, each copied from the best-matching haplotype in a pool of donor populations (*36*). In populations where high levels of differentiation are mainly due to genetic drift, recipients tend to copy most DNA chunks from donors belonging to their own or closely related groups, obscuring ancestry shared with other groups (*37*). As outlined by van Dorp *et al.* (*37*), if recipients are prevented from finding their best-matching haplotypes inside their own group, the effects of drift are attenuated and genetic relationships to other groups can be better evaluated. Mimicking this approach, we did not allow pastoralists and peripatetics from southwestern Angola and northwestern Namibia to copy haplotypes from each other to evaluate whether their genetic distinctiveness could be attributed to differences in their ancestry composition before recent isolation. Under these conditions, the Kwepe, Twa, and Kwisi display very similar copying profiles that are clearly differentiated from the profiles of their pastoralist (Fig. 3, C and D) and agropastoralist (fig. S12, C and D) neighbors. The profile of the Tjimba is close but not identical to the other peripatetics, while the Damara fully align with the Bantu-speaking pastoralists (Fig. 3, C and D, and fig. S11, C and D). Comparisons of profile differences between each group and representative pastoralist (Kuvale) and agropastoralist (Ovimbundu) populations further show that peripatetic groups have decreased amounts of Bantu and West African–related ancestries, while copying elevated numbers of haplotypes from groups carrying southern African forager ancestry and, to a lesser extent, the Mbuti rain forest hunter-gatherers and several East African groups (Fig. 3, A and B, and fig. S12, A and B). This finding highlights the impact of differential admixture with pre-Bantu populations and suggests that drift and inbreeding were not the only factors influencing their genetic differentiation.

**Fig. 4.**
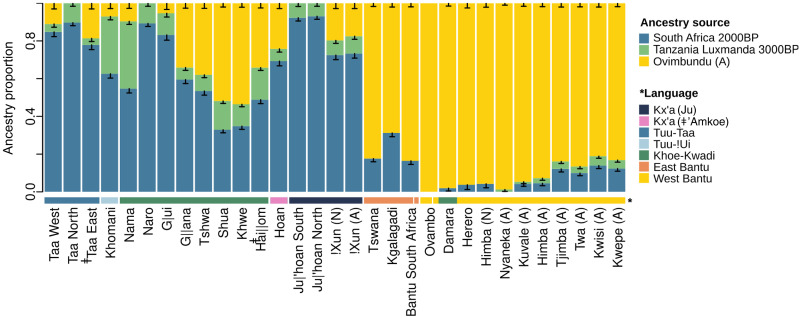
Ancestry proportions estimated with qpAdm in southern African populations. SEs (black bars) were calculated with a weighted block jackknife. BP, before the present.

To further assess the role of pre-Bantu admixture while including ancient individuals as potential sources (*16*), we used qpAdm (*38*). By testing the fit of different mixture models, we confirm that the peripatetic peoples (Kwepe, Kwisi, Tjimba, and Twa) diverge from their neighbors (Himba, Kuvale, and Nyaneka) by displaying higher amounts (10 to 14%) of southern African forager ancestry [here represented by an ancient South Africa 2000 before the present (B.P.) genome] and a detectable contribution (4 to 5%) from East Africa, best matched by an ancient genome retrieved from a pastoral context in Tanzania (Luxmanda 3100 B.P.) (Fig. 4, text S2, fig. S13, and tables S3 and S4).

Our inference of the relative order of mixing of the Bantu-, autochthonous southern African–, and East African–related ancestries in southwestern Angola and northwestern Namibian groups, based on ancestry covariances (*39*), indicates that the first admixture event involved southern African– and East African–related ancestries (table S5), in accordance with the archaeological data suggesting that pastoralism was introduced from eastern into southern Africa before the Bantu expansion (*40*). While we could not obtain reliable estimates for this admixture event (text S3), we detected signals of admixture between Bantu and the previously admixed pre-Bantu ancestries dating to ~600 to 1100 years ago by using Wavelets, GLOBETTROTER, and ALDER (fig. S14 and tables S5 to S7). As these estimates postdate the arrival of the first Bantu speakers in southern Africa (~1.8 to 1.5 ka ago), they may either point toward a delayed colonization of areas in and around the Namib Desert or to a delayed onset of admixture between resident and incoming populations. Alternatively, multiple pulses of admixture might have occurred at different times, in which case the inferred dates would be intermediate between the oldest and the most recent admixture events (*41*).

Similar to the Angolan Namib, other areas where languages of the Khoe-Kwadi family are spoken today were strongly shaped by contact and display highly variable amounts of southern African–, East African–, and Bantu-related ancestries (Fig. 4). To reconstruct the contact histories of the Khoe-Kwadi following their arrival to southern Africa, we carried out local ancestry decomposition (*42*) and analyzed the population relationships within each of the three major settlement layers of southern Africa (figs. S15 to S18).

On the basis of PCA projections, we found that the East African ancestry identified in the genomes of Khoe-Kwadi speakers and other southern Africans is related to pastoralist groups clustering around the ancient Tanzania Luxmanda individual (3100 B.P.) (fig. S16). Some Nama and ǂKhomani individuals are additionally related to East African groups with high amounts of Eurasian ancestry, likely due to admixture with Europeans during colonial times.

**Fig. 5.**
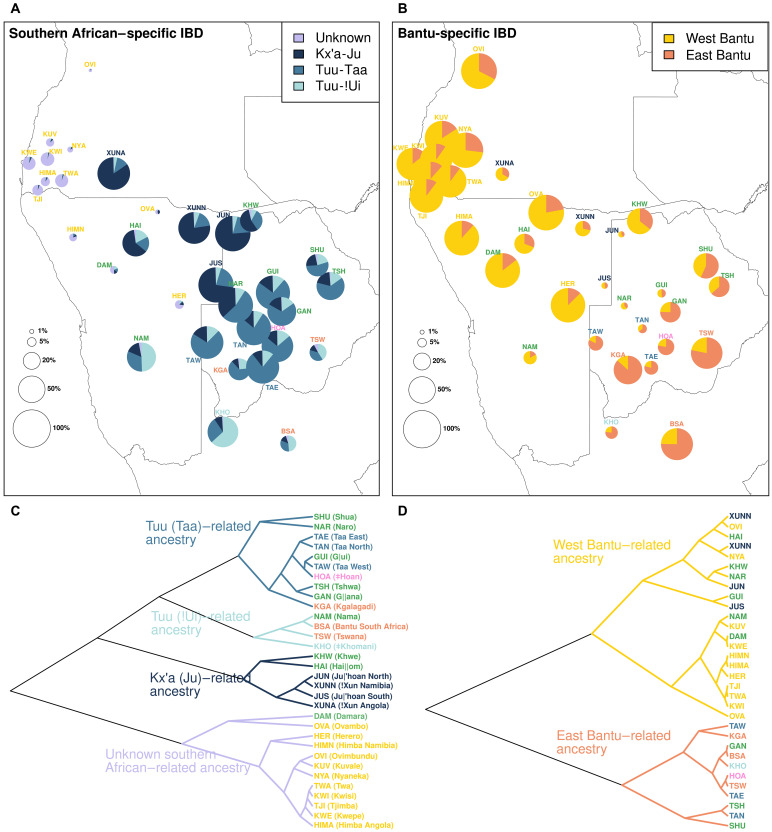
Summary of the ancestry-specific IBD sharing. The pie charts represent the proportion of autochthonous southern African-related (i.e., neither Bantu- nor East African–related) (**A**) and Bantu-related (**B**) IBD shared on average between each target group and the pools of populations identified in the figure legend. The category “Unknown” represents southern African–related IBD shared with the peripatetic groups from the Angolan Namib. The percentage of southern African (A) and Bantu (B) ancestry within each target group is represented by the total area of the pie chart. (**C**) and (**D**) show a UPGMA clustering of all target groups according to their shared southern African– and Bantu-related IBD, respectively. Detailed results for every population pair are shown in fig. S20. Population abbreviations according to (C).

Ancestry-specific PCA (figs. S17 and S18) and clustering analysis based on average pairwise differences (fig. S19) further show that southern African– and Bantu-related ancestries of Khoe-Kwadi groups are highly heterogeneous and mirror the genetic composition of their neighbors. This pattern becomes especially clear when the local ancestry information is combined with IBD inferences to obtain southern African– and Bantu-specific IBD sharing (Fig. 5 and fig. S20). For example, the Khwe from the northern Kalahari Basin fringe have southern African– and Bantu-related ancestries that are similar to those of !Xun foragers and Southwest Bantu agropastoral groups living in the same area (Fig. 5 and fig. S20). To their south, Khoe speakers from the Central Kalahari share southern African–related ancestry with the neighboring Taa and ǂHoan, and Bantu-related ancestry with local East Bantu speakers (Fig. 5 and fig. S20). The southern African– and Bantu-related ancestries of the Khoekhoe-speaking Nama reflect their migration history along the Atlantic coast of southern Africa. While their southern African–related ancestry resembles that of the ǂKhomani, who inhabit the southernmost areas of the Kalahari, their Bantu ancestry is similar to that of Southwest Bantu–speaking groups from northwestern Namibia (Fig. 5 and fig. S20). As the Nama are known to be a branch of Khoekhoe-speaking groups who migrated northward from South Africa (*43*), it is likely that they first acquired their southern African–related ancestry in the South and admixed with Bantu populations only later after reaching Namibia.

**Fig. 6.**
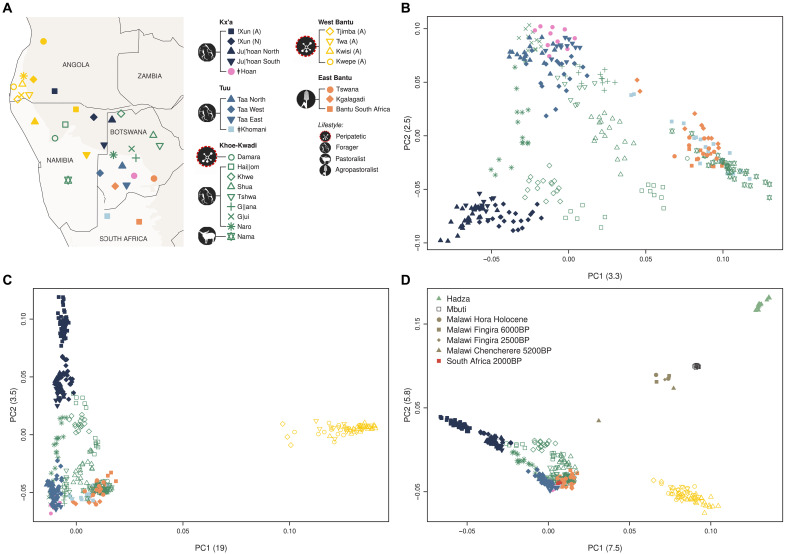
PCA of southern African–related ancestry. (**A**) Map showing approximate sample locations. The lightest background color shows the desert and xeric shrublands biome. (**B** to **D**) PCA built with previously published southern African individuals (see references in table S2) that display <10% of missing data after masking the non–southern African ancestries (B), additionally including Angolan individuals (C), and other relevant modern (see references in table S2) or ancient groups (D) (*16*, *17*). The axis labels include the eigenvalue (in parentheses) for each PC.

In the Angolan Namib, the formerly Kwadi-speaking Kwepe and the other peripatetic groups all share Bantu-related ancestry with the southwestern Bantu pastoralists that surround them (Fig. 5 and figs. S19 and S20). However, their southern African–related ancestry does not match any of the major ancestry components that have previously been described in southern Africa (Fig. 5 and figs. S19 to S21): Despite sharing large amounts of southern African–related IBD segments among themselves, the peripatetics stand out for their lack of IBD sharing with present-day southern African forager groups (Fig. 5 and figs. S20 and S21), suggesting that their southern African–related ancestry resulted from admixture with a deeply divergent unsampled group. The same ancestry is also found in other groups from southwestern Africa, including the Damara from Namibia, but the detected frequencies are much lower than in the Angolan peripatetics (Fig. 5 and figs. S20 and S21). The uniqueness of this previously undetected genetic component [henceforth called Khoisan (KS)–Namib] is also supported by a PCA undertaken with the EMU (expectation-maximization PCA for Ultra-low Coverage Sequencing Data) approach (*44*). This approach, which allows for the detection of population structure even with high levels of missing data, shows that KS-Namib can be readily separated from all known major African ancestries, including the southern African–related component identified in ancient (8100 to 2500 B.P.) hunter-gatherers from Malawi (Fig. 6, C and D). A reconstruction of the topology of southern African–related ancestries based on genealogical concordance (*3*, *45*) further shows that the separation of KS-Namib predates the separation of other southern African ancestries, indicating a deep divergence of this component (text S4 and table S8). An early split is further suggested by estimates of divergence time between pairs of populations, showing that the separation of KS-Namib is 13 to 44% older than the split times of the southern African–related ancestries associated with Kx’a (Ju)–, Tuu (Taa)–, and Tuu (!Ui)–speaking populations, assuming an effective population size (*Ne*) of 20,000 individuals (text S4 and table S9) (*3*). Together, these results suggest that the Angolan Namib Desert and its surrounding areas preserve the legacy of an extinct, deeply divergent human group with no close matches in extant populations from within and outside southern Africa.

## DISCUSSION

The Angolan Namib Desert provides an invaluable framework to examine the history and consequences of contact and admixture between different migratory waves into the wider region of southern Africa. Despite being culturally dominated by Southwest Bantu–speaking cattle herders, the area is remarkable for the presence of several impoverished groups with a peripatetic way of life, who have attracted a considerable degree of ethnographic interest (*21*, *27*, *43*, *46*, *47*).

Our results highlight the heterogeneity of the population landscape of the Angolan Namib by showing that, despite their high amounts of Bantu ancestry (~80%), all sampled peripatetic groups display elevated levels of an eastern African ancestry and a previously undocumented southern African–related component (KS-Namib) (Figs. 4 to 6). The co-occurrence of the two pre-Bantu ancestries among all peripatetic groups hints at a complex contact and admixture history. Because the eastern African component has also been detected in Khoe-speaking groups of the Kalahari Basin (Fig. 4), it is likely that it was introduced to southwestern Angola by the ancestors of the present-day Kwepe as part of the Kwadi branch of the Khoe-Kwadi pastoral dispersal. By contrast, KS-Namib has a more restricted distribution, being especially common in the Angolan Namib and appearing in residual amounts in other groups of southwestern Africa, including the Damara from Namibia who are historically linked to a peripatetic way of life (Fig. 5). This distribution suggests that KS-Namib was more likely associated with a resident foraging population of southwest Africa than with migrants from elsewhere.

While at present no hunter-gatherers resembling Kx’a- and Tuu-speaking groups exist in the Angolan Namib, an early account by 16th-century traveler Duarte Pacheco-Pereira states that the areas around the Kuroka River mouth were then inhabited by nomadic groups who lived on fishing and built houses from whale ribs that they covered with seaweed (*48*). This description is reminiscent of coastal foragers often referred to as “Strandlopers” who once lived near the coast in Namibia and South Africa but went extinct during the 19th century (*49*). Although historical records note them to be Khoekhoe-speaking, their culture differed from the herding groups further inland, and their origins may ultimately trace to resident hunter-gatherer groups, as suggested by the long history of maritime foraging in southern Africa (*50*). An ancient, prepastoral origin of southern African marine foragers was recently supported by genome-wide data from a 2241- to 1965-ka-old skeleton excavated at St. Helena Bay on the coast of South Africa (*16*, *51*). However, the genetic profile of this individual is close to contemporary Tuu-speaking hunter-gatherers from inland areas of southern Africa and not to the KS-Namib component (Fig. 6). Hence, it is likely that only studies of ancient DNA from the southwestern coast of Angola will be able clarify the ancestral relationships between KS-Namib and extinct forager populations. While the archaeological record for the Angolan Namib is sparse, it is possible that prehistoric human remains associated with this ancestry may be recovered around shell midden deposits and vestiges of coastal settlements that have been reported in areas close to the Kuroka River mouth (*52*, *53*).

An ancient occupation of the Namib coast is also supported by oral stories describing an encounter between the Kwadi-speaking Kwepe and resident peoples who had no fire and ate raw fish on the beach (*27*, *52*, *54*). Some anthropologists have equated these resident fishermen with the ancestors of the Kwisi and Twa, because of their present socioeconomic marginalization and historically documented association with hunting and gathering, which contrasts with the Kwepe’s higher reliance on small-scale pastoralism (*27*, *52*, *54*).

However, while our results show that present-day Twa and Kwisi can be genetically separated from the Kwepe (figs. S4 and S6), the three groups are virtually indistinguishable once the effects of genetic drift are attenuated (Fig. 3 and fig. S12). This pattern suggests that all extant peripatetic groups are equally related to different ancestries, thus challenging any attempts to establish continuity between specific modern populations and ancient foragers, beyond ethnographic considerations. The microcosmos of the Angolan Namib can therefore be considered a highly stratified polyethnic system (*55*) where groups with different genetic and ethnolinguistic backgrounds admixed but maintained sharp divisions based on their socioeconomic status.

This contact profile of southwestern Angola is remarkably similar to other areas of southern Africa where Khoe-Kwadi–speaking migrants encountered resident populations with different linguistic and genetic legacies. A defining characteristic of all these areas is an admixture history, which starts with the fusion between an eastern African ancestry and different resident southern African forager components, later followed by various degrees of admixture with Bantu speakers from the western and eastern streams of the Bantu migrations (*19*). Considering the available genetic, linguistic, and archaeological data (*19*, *40*, *56*), we hypothesize that proto–Khoe-Kwadi speakers split in the northwestern Kalahari, contrary to previous proposals that assume a southwestward movement from an intermediate homeland in northeastern Botswana (*43*, *57*). Following their split, the Khoe-Kwadi diverged into different groups migrating into specific contact areas (figs. S1 and S22): Khoekhoe speakers moved south along the Atlantic coast, encountering !Ui-speaking groups; Kalahari Khoe speakers took an eastward route where they encountered Ju speakers in the northern Kalahari, and Taa and ǂHoan speakers in the central Kalahari; and Kwadi speakers migrated toward southwestern Angola, arriving in areas inhabited by a now extinct foraging group associated with the KS-Namib component. More recently, most Khoe-Kwadi–speaking peoples were further affected by East and West Bantu–speaking populations, adding to their diverse genetic makeups.

Together, our results show that contact areas associated with the confluence of different migratory waves can harbor the ancestry of vanished groups predating the arrival of food production in Africa. While the full diversity and geographical extension of these early foragers may ultimately be revealed by ancient DNA, detailed studies of highly admixed small-scale communities can still provide unique opportunities to probe the deep genetic structure of the continent.

## MATERIALS AND METHODS

### Sample information

This study includes a total of 208 samples from nine ethnic groups of southwestern Angola. The sample size, linguistic affiliation, subsistence pattern, and sampling locations for each group are summarized in table S1. Samples from the Nyaneka and Ovimbundu were collected as described in (*58*), and samples from the remaining populations (!Xun, Kwepe, Kwisi, Twa, Tjimba, Himba, and Kuvale) were collected as described in (*22*). Written informed consent was obtained from all participants. This study was developed in the framework of a collaboration between the Portuguese-Angolan TwinLab established between CIBIO/InBIO and ISCED (Instituto Superior de Ciências de Educação)–Huíla Angola, and has the ethical clearance of ISCED and the CIBIO/InBIO–University of Porto boards and the support and permission of the Provincial Governments of the Namibe and Kunene.

### Genotyping and quality control

All sampled individuals were genotyped on the Affymetrix Axiom Genome-Wide Human Origins Array (*59*). The data generated in this work were analyzed together with data from 54 African populations (550 individuals) previously genotyped on the same array (table S2) (*2*, *59*, *60*), after filtering out single-nucleotide polymorphisms (SNPs) with a missing rate higher than 10%, SNPs with deviations from Hardy-Weinberg equilibrium (i.e., *P* value <0.001 in more than two populations), and SNPs from nonautosomal markers. These filters yielded a final set of 607,761 SNPs. No individuals had missing rates above 10%. We excluded from the analyses 37 individuals from Angola and 25 individuals from elsewhere because of cryptic relatedness. Specifically, we removed one individual from each pair that exhibited a proportion of IBD higher than 0.2, computed in PLINK v1.9 as P(IBD = 2) + 0.5 × P(IBD = 1) (*34*). We additionally generated a dataset pruned for linkage disequilibrium (LD) with PLINK v1.9 (*34*), removing SNPs with *r*^2^ > 0.4 in 200-kb windows, shifted at 25 SNP intervals. The pruned dataset includes a total of 350,719 SNPs. These datasets were additionally merged with ancient samples from published sources (*16*, *61*, *62*).

### Phasing

All present-day samples analyzed were jointly phased with Beagle 4.1 (*63*) using the HapMap genetic map available from the Beagle website (https://faculty.washington.edu/browning/beagle/beagle.html).

### Population structure analyses

#### Genotype-based

PCA was computed using the smartpca software from EIGENSOFT 6.0.1 (*31*), without exclusion of outliers (“numoutlieriter: 0”). ADMIXTURE v1.3 (*32*) was used to estimate ancestry proportions originating from *k* ancestral populations, with *k* ranging from 2 to 16, and applying a cross-validation procedure, for a total of 15 independent runs. In addition, we used DyStruct v.1.1.0 (*64*) to identify shared ancestry with relevant ancient individuals from East and southern Africa, taking into account the individual’s archaeological age and assuming a generation time of 29 years (*65*). The DyStruct analysis included only a subset of the present-day groups used in the ADMIXTURE analysis, and the Angolan groups were additionally downsampled to lower the impact of large sample sizes in clustering analysis. We performed six independent runs, using two to six ancestral populations. The ADMIXTURE and DyStruct results were plotted with pong (*66*). These analyses were carried out using the LD-pruned dataset.

#### Haplotype-based

We used refinedIBD v.17Jan20.102 (*33*) to identify IBD blocks shared between individuals and homozygous-by-descent (HBD) blocks shared within each individual. Throughout the text, we refer to the combined IBD and HBD blocks simply as IBD. Blocks within a 0.6-cM gap were merged using the software merge-ibd-segments v.17Jan20.102 (https://faculty.washington.edu/browning/refined-ibd), allowing one inconsistent genotype between the gap and block regions. The IBD blocks were then partitioned into three length categories (1 to 5, 5 to 10, and more than 10 cM) to investigate IBD sharing across different time periods (*67*, *68*). The average total length shared within and between populations was summarized for each category using networks built with the R package ggraph v2.0.5, and the distribution of total lengths shared within populations was inspected with the R package vioplot v.0.3.7. We used a Wilcoxon rank sum test (wilcox.test in R) to compare differences in the mean lengths of shared IBD segments between two groups.

We estimated changes in the effective population size (*Ne*) though time based on IBD blocks of at least 2 cM shared within populations, using the software IBDNe v.23Apr20.ae9 (*35*). The *Ne* is shown for generations 4 to 50, which corresponds to the time period for which IBD segments are informative when using SNP array data (*35*).

RoHs were identified in PLINK v1.9 (*34*) using the LD-pruned dataset and default parameters: sliding windows of 50 SNPs, an RoH minimum length of 1 Mb, five missing genotypes and one heterozygote allowed per window, and a scanning window hit rate of 0.05 required for an SNP to be eligible for the RoH.

We used CHROMOPAINTER v2 (*36*) to infer a “painting” or copy profile for individuals from southwestern Angola and northwestern Namibia, based on two different sets of donor and recipient populations. These were chosen to test whether all groups share a common ancestor before any recent isolation. In the first set, pastoralists and peripatetics from southwestern Angola and northwestern Namibia (recipients) were only allowed to copy haplotypes from Bantu-speaking agropastoralists (Nyaneka, Ovimbundu, and Ovambo) or more distantly related African groups, therefore minimizing differences in copy profiles caused by recent genetic drift (Fig. 3). In the second set, agropastoralists were included as recipients and excluded from the donors; hence, all individuals from southwestern Angola and northwestern Namibia could only copy haplotypes from distantly related African groups (fig. S12). To account for the impact of uneven donor sample sizes in the resulting copy vectors, each analysis was run three times with a random sample of five individuals per donor population. We used all of the available individuals for populations with less than five individuals.

We initially estimated the mutation emission and switch rate parameters using 10 iterations of the expectation-maximization (EM) algorithm and a subset of chromosomes (1, 5, 10, 15, and 22). The inferred parameters were averaged by chromosome (taking into account their number of SNPs) and then by individuals. We fixed these parameters and performed an additional CHROMOPAINTER run for all chromosomes.

The copy profiles generated for each recipient individual under each analysis were displayed in coancestry matrices that represent the average copy profiles of three runs. The difference between the average copy profiles of any pair of populations x and y, under each set, was quantified using the total variation distance (TVDxy) (*37*, *69*). A nonmetric multidimensional scaling (MDS) was carried out in R, using the function isoMDS.

### Ancestry modeling with qpAdm

We used qpAdm v.650 (*59*) to test one-wave, two-wave, and three-wave admixture models for each southern African population and to estimate their respective admixture proportions. The tests were conducted using the same reference and source populations as in (*16*)—chosen to capture major strands of ancestry in Sub-Saharan Africa, as well as using a modified set of reference and source populations that is best suited for populations of southern Africa (text S2 and fig. S13). We applied a “rotating” strategy (*16*, *70*) for each of the two sets of populations in which a defined number of sources (one, two, or three) was selected iteratively from a source pool, while all the other populations in the set were used as references. We rejected models if their *P* values were lower than 0.05, if there were negative admixture proportions, or if the SEs were larger than the corresponding admixture proportion. As more than one model was often accepted per population (tables S3 and S4), we display the results according to different criteria (text S2 and fig. S13).

### Local ancestry inference

Local ancestry inference was carried out using RFMix v2 (*42*) (https://github.com/slowkoni/rfmix; v2; accessed 3 August 2020) for all southern African individuals. Equal-size samples (*n* = 13) of Yorubans, Somalis, and southern African individuals (8 Ju|’hoan North, 5 Taa West) that occupy the rightmost position in PC1 (Fig. 1, B and C) were used as training sources to capture ancestry related to the Bantu expansion, the eastern African pastoral migration, and the indigenous southern African hunter-gatherer stratum, respectively. We ran RFMix with three iterations and the option “reanalyze-reference” to account for any admixture in the references, used a minimum of five reference haplotypes per tree node, and assumed 25 generations since the admixture event.

### Admixture timing

The relative order of mixing of different ancestries in admixed populations from southwestern Angola and northwestern Namibia was inferred using the Admixture History Graphs (AHG) approach (*71*). In an admixed population with two ancestry components (A and B) that later receives a third component (C) via admixture, the ancestry proportions of A and B will covary with C, but the ratio of A and B throughout the population will be independent from C. The AHG approach involves estimating the covariance of the frequencies of A/B with C, A/C with B, and B/C with A, across all individuals in the population, and identifying the configuration that produced the smallest absolute value of the covariance estimate. The individual ancestry proportions used in this test were those inferred by RFMix.

The dating of the admixture events was obtained via the wavelet-transform analysis (*39*), which uses the width of ancestry blocks identified by RFMix to determine the time since admixture by comparing the results to simulations (*39*, *71*). In this analysis, we assume the order of events as inferred by the AHG approach.

We additionally estimated admixture events with GLOBETROTTER (*72*). First, to minimize any noise in the admixture inference caused by outlier individuals, we performed a fineSTRUCTURE analysis (*36*), which hierarchically divides individuals into genetically homogeneous groups. By comparing those groups with the ethnic label of each individual, we identified a total of 32 outlier individuals (3 Kwepe, 3 Kwisi, 5 Twa, 3 Tjimba, 4 Himba, 8 Kuvale, and 5 Xun), which we excluded from the admixture analyses. Then, we performed another CHOMOPAINTER run in which both recipient and surrogate individuals were “painted” by the same set of donors as in Fig. 3. Last, we ran GLOBETROTTER, using 10 painting profiles per individual and the coancestry matrix for the total length of haplotype sharing obtained with CHROMOPAINTER. All donor populations were included as possible surrogates (i.e., sources of admixture). Briefly, GLOBETROTTER dates a maximum of two admixture events based on the decay of LD versus genetic distance among the segments copied from pairs of surrogate populations and infers the sources of admixture as a linear combination of the DNA of the sampled groups. As recommended by the authors (*72*), we performed this analysis with and without a “NULL” individual to evaluate the consistency of the estimates. We ran 50 bootstrap iterations to infer confidence intervals for the date estimates.

For comparison, we also estimated the time of admixture between Bantu and southern African ancestries based on the exponential decay of LD using ALDER v1.03 (*73*) with default settings and using as references the Yoruba and Ju|’hoan North. All admixture dates are presented assuming a generation time of 29 years (*65*).

### Ancestry-specific analyses

For the ancestry-specific analyses, all ancestries except the one of interest were masked (treated as missing) in each haploid genome. In addition, we masked all positions within each haploid genome for which the marginal probability returned by RFMix was smaller than 1, thus excluding any parts of the genome where the ancestry assignment was ambiguous. We first confirmed the validity of the local ancestry inference and masking procedures by computing a PCA on the training sources used in RFMix (Yoruba, Somali, and the least admixed Ju|’hoan North and Taa West individuals) and projecting on it all target southern African haploid genomes after masking (fig. S15). The PCA was carried out with smartpca (*59*) using the lsqproject option. Note that to keep standard file format requirements while allowing for unequal missingness in the two haploid genomes composing an individual, we treat each haploid genome as an individual in the PCA projections, therefore displaying twice the number of diploid individuals. Because all genomes with less than 95% missing data for a given ancestry overlap with the expected source population, we used this cutoff for the ancestry-specific PCA.

The PCA targeting East African–specific ancestry was built with unmasked genomes from present-day East African populations, excluding Bantu-speaking groups and the Luo (who display a Bantu-related profile) and filtering out SNPs with a missing rate higher than 10% (fig. S15). Southern African individuals with less than 95% missing data after masking the non–East African ancestry were then projected, together with previously published ancient genomes from East Africa (fig. S16). The PCA of the autochthonous southern African ancestry was built with a subset of southern African individuals displaying <25% of missing data after masking the non–southern African ancestries and the additional removal of SNPs with a missing rate higher than 15%. The remaining southern African individuals having between 25 and 95% missing data after masking were projected (fig. S17). Similarly, the PCA of Bantu-specific ancestry was constructed using southern African individuals, as well as Bantu-speaking groups from East Africa, having <25% of missing data after masking the non-Bantu ancestries, with the additional removal of SNPs with a missing rate higher than 15%. The remaining individuals having between 25 and 95% missing data after masking the non-Bantu ancestries were projected (fig. S18).

PCA was additionally carried out with an alternative method (EMU-EM-PCA for Ultralow Coverage Sequencing Data), designed to handle high missingness in genetic datasets (Fig. 6) (*44*). This analysis was computed on two eigenvectors, using southern African individuals that have <10% of missing data after masking.

We calculated ancestry-specific pairwise differences and constructed heatmaps with built-in dendrograms using R (https://R-project.org/). Specifically, we used the function hclust, the hierarchical clustering method complete linkage, and a correlation matrix to compute the distance between both rows and columns of the pairwise distance matrix, represented in R by as.dist[1-cor(x)] (fig. S19).

Last, we combined the information on IBD sharing between southern African individuals with their local ancestry profiles to obtain ancestry-specific IBD segments. For this analysis, we used the raw IBD blocks before the merging step, because that step would lead to loss of information about the haplotype of origin. While an IBD block might be a mosaic of more than one ancestry, the ancestry along two haplotypes that are identical by descent should, in theory, match. Yet, in practice, mismatches can be found if the RFMix inference is not perfect. Thus, we excluded from this analysis IBD blocks whose ancestry along both haplotypes is inconsistent for more than 25% of the corresponding IBD length (in centimorgans). For each of the remaining IBD blocks shared between two (haploid) individuals, we recorded the length of the block (in centimorgans) associated with each specific ancestry and then computed the sum of all lengths per ancestry. The average sum of IBD lengths (in centimorgans) from a specific ancestry and the average sum of IBD lengths excluded because of inconsistent ancestry assignments are displayed for each pair of populations using stacked barplots (fig. S20). This procedure, made available in https://zenodo.org/record/8138890 and https://github.com/sroliveiraa/asIBD, was applied separately for IBD blocks belonging to different length categories (1 to 5, 5 to 10, and more than 10 cM).

We additionally summarized the results for length category 1 to 5 cM by computing the average IBD sharing between each group and pools of populations representing the genetic diversity of southern African and Bantu-related ancestries. These comprise West Bantu– and East Bantu–related ancestries, as well as southern African ancestries represented by Kx’a-Ju–, Tuu-Taa–, and Tuu-!Ui–speaking populations. We further included in the comparisons an “unknown” component to account for the southern African–related ancestry shared with peripatetic groups from the Angolan Namib (Fig. 5, A and B). A UPGMA (unweighted pair group method with arithmetic mean) cladogram representing the Euclidean distance between proportions of IBD sharing between each population and these major groups was built with the function upgma of the R package poppr (Fig. 6, C and D).

### Population topology and divergence time inferences

We used genealogical concordance (*3*, *45*, *74*) to infer the population topology of the main southern African–related ancestries, including the previously undetected KS-Namib ancestry (text S4). This approach is based on randomly sampling a single gene copy from each of four populations, therefore avoiding the effect of genetic drift within populations (*3*), and can be applied to ancestry-specific (masked) data.

For each combination of three populations and the Chimpanzee (Chimpanzee, P_1_; P_2_, P_3_), we randomly sampled one nonmissing allele per population at each site and calculated the number of concordant *N*_conc_ (AABB) and discordant *N*_disc_ (ABBA or ABAB) topologies. We then computed the excess of putatively concordant topologies over the second most frequent discordant category (disc1) as in Schlebusch *et al.* (*3*)

$$C=\frac{N_{\mathrm{conc}}-N_{\mathrm{disc}1}}{N_{\mathrm{conc}}+N_{\mathrm{disc}1}}$$ (1)

We used a block jackknife procedure, in which one of 50 contiguous blocks with the same number of informative SNPs was excluded iteratively, to obtain SEs

$${\mathrm{SE}}_{\mathrm{jack}}=\sqrt{\frac{n-1}{n}\times \sum {\left(Ci-\bar{C}\right)}^{2}}$$ (2)

where *n* is the number of blocks, *Ci* represents each of the *C* values obtained after excluding one block, and $\bar{C}$ is the mean of the *C* values. *Z* scores were calculated on the basis of the number of SEs the statistic deviates from zero

$$Z=\frac{C-0}{{\mathrm{SE}}_{\mathrm{jack}}}$$ (3)

In all tests, P_1_ includes all peripatetic individuals, P_2_ is represented by Kx’a-Ju–speaking individuals, and P_3_ is represented by Tuu-Taa– or Tuu-!Ui–speaking individuals. To minimize the influence of ascertainment bias [see (*3*)], we performed each test using the intersection of SNPs (i) that are polymorphic in each population (i.e., P_1_, P_2_, and P_3_) and (ii) that have a minor allele frequency > 10% and more than 10 alleles per population. In addition, because the chance of finding a polymorphism depends on the sample size and the amount of nonmissing alleles in each population is quite variable, we first downsampled individuals so that, on average, there was a similar number (10 to 12) of nonmissing alleles per SNP per population. Moreover, we repeated all tests excluding SNPs discovered in a Juǀ’hoan North individual [San ascertainment; (*59*)] so that any remaining ascertainment is expected to equally affect the southern African–related ancestry of each population involved in the test.

We additionally used genealogical concordance to infer divergence times between pairs of populations (*3*, *45*). For this purpose, at each site, we randomly sampled two alleles from one population (in-group) and one allele from another population (out-group), and extracted the Chimpanzee allele. SNPs with informative configurations were then classified as concordant or discordant, and the internode time *T* (which is proportional to the total divergence time between the two populations) was estimated as in Schlebusch *et al.* (*3*)

$$\hat{T}=-\log\left(\frac{3-3P_{\mathrm{conc}}}{2}\right)$$ (4)

where *P*_conc_ is the proportion of concordant genealogies. Confidence intervals were computed with a maximum likelihood framework (*3*, *45*, *74*). We converted *T̂*, which is measured in units of coalescent effective population size, into chronological dates by assuming *Ne* values ranging from 1000 to 20,000 for KS-Namib, *Ne* values of 20,000 for the remaining in-groups, and a generation time of 29 years (*65*).

## Supplementary Materials

### This PDF file includes:

Figs. S1 to S22 Texts S1 to S4 Legends for tables S1 to S9 References
